# The genome sequence of the malaria mosquito, *Anopheles funestus*, Giles, 1900

**DOI:** 10.12688/wellcomeopenres.18445.1

**Published:** 2022-11-25

**Authors:** Diego Ayala, Ousman Akone-Ella, Pierre Kengne, Harriet Johnson, Haynes Heaton, Joanna Collins, Ksenia Krasheninnikova, Sarah Pelan, Damon-Lee Pointon, Ying Sims, James Torrance, Alan Tracey, Marcela Uliano-Silva, Katharina von Wyschetzki, Jonathan Wood, DNA Pipelines collective, Shane McCarthy, Daniel Neafsey, Alex Makunin, Mara Lawniczak

**Affiliations:** 1 MIVEGEC, IRD, Montpellier, 34394, France; 2 ESV-GAB, Centre Interdisciplinaire de Recherches Médicales de Franceville (CIRMF), Franceville, BP 769, Gabon; 3 Scientific Operations, Wellcome Sanger Institute, Hinxton, CB10 1SA, UK; 4 CSSE, Auburn University, Auburn, AL, 36849, USA; 5 Tree of Life, Wellcome Sanger Institute, Hinxton, CB10 1SA, UK; 6 Department of Genetics, University of Cambridge, Cambridge, CB2 3EH, UK; 7 Department of Immunology and Infectious Diseases, Harvard T.H. Chan School of Public Health, Boston, MA, 02115, USA; 8 Infectious Disease and Microbiome Program, Broad Institute, Cambridge, MA, 02142, USA

**Keywords:** Anopheles funestus, African malaria mosquito, genome sequence, chromosomal inversions

## Abstract

We present a genome assembly from an individual female *Anopheles funestus* (the malaria mosquito; Arthropoda; Insecta; Diptera; Culicidae). The genome sequence is 251 megabases in span. The majority of the assembly is scaffolded into three chromosomal pseudomolecules with the X sex chromosome assembled. The complete mitochondrial genome was also assembled and is 15.4 kilobases in length.

## Species taxonomy

Animalia; Arthropoda; Insecta; Diptera; Culicidae; Anophelinae; Anopheles; *Anopheles funestus*; Giles, 1900 (NCBI txid:62324).

## Background

The mosquito *Anopheles funestus* is one of the major malaria vectors in Sub-Saharan Africa ^1^. Although it can have a sparse and patchy distribution, this mosquito species is present nearly everywhere across the continent from the savannahs of West-Africa, the rainforest of Central Africa, through the dry valleys of East Africa until the Red Island of Madagascar ^2^. *Anopheles funestus* breeds in natural and artificial, permanent or semi-permanent water bodies such swamps or rice fields. It is a member of a species group containing at least thirteen species, among which it is the most medically important species ^3^. Its prominent role in the transmission of the malaria parasites is due to its close relation to humans, which provide shelters, breeding sites, and blood meals ^1^. Although this association makes it highly susceptible to vector campaigns such as indoor residual spraying (IRS) and insecticide treated nets (ITNs), this mosquito species has become resistant to multiple insecticides in many parts of Africa ^4^. Therefore, any program aiming at eradicating malaria cannot ignore this species. 

At the genetic level, *Anopheles funestus* has been historically neglected in comparison to the members of the *Anopheles gambiae* complex. Multiple studies using genetic markers, such as microsatellites, chromosomal inversions, or DNA sequences have revealed the extraordinary genetic and inversion polymorphism of this species. This genetic richness is likely to underlie its ecological plasticity ^5^, its ability to overcome insecticide pressures ^6^, and incipient speciation ^7^. The first complete genome draft of this mosquito appeared in 2015, originating from a colony derived from wild individuals collected in Mozambique (Fumoz) ^8^. Later, the quality of the reference genome for Fumoz was improved using long read sequencing from multiple individuals ^9^. Here, as part of the *Anopheles* Reference Genomes Project (PRJEB5169), we present a chromosomally complete genome sequence for *Anopheles funestus*, based on a single female specimen from La Lopé, Gabon.

## Genome sequence report

The genome was sequenced from a single female *Anopheles funestus* collected from La Lopé, Gabon (-0.187, 11.611). A total of 56-fold coverage in Pacific Biosciences single-molecule long reads (N50 10.684 kb) and 68-fold coverage in 10X Genomics read clouds were generated. Primary assembly contigs were scaffolded with chromosome conformation Hi-C data from a female sibling. Manual assembly curation corrected four missing joins or misjoins, reducing the scaffold number by 0.6%.

**Figure 1.**
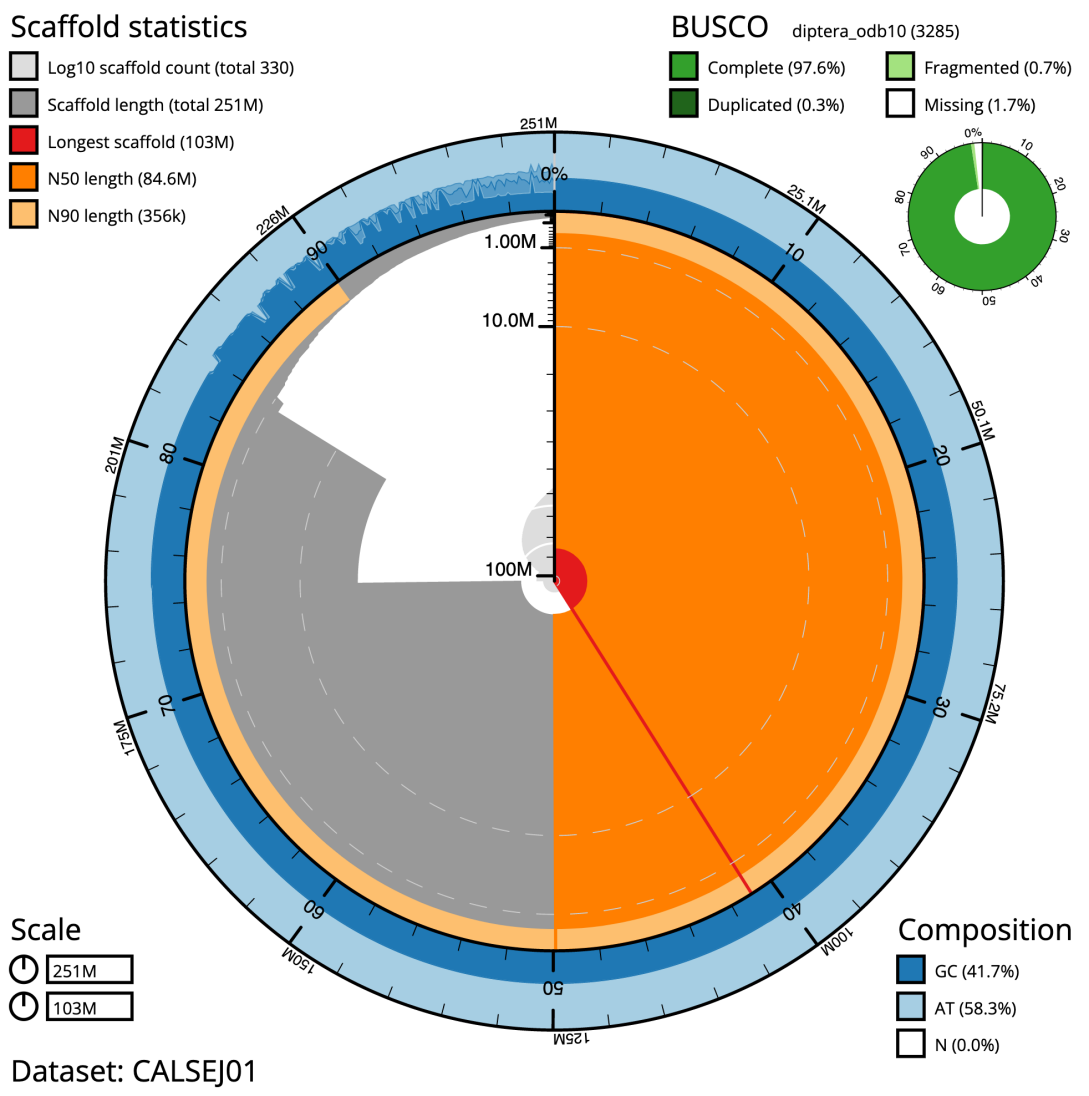
Genome assembly of *Anopheles funestus*, idAnoFuneDA-416_04: metrics. The BlobToolKit Snailplot shows N50 metrics and BUSCO gene completeness. The main plot is divided into 1,000 size-ordered bins around the circumference with each bin representing 0.1% of the 250,713,484 bp assembly. The distribution of chromosome lengths is shown in dark grey with the plot radius scaled to the longest chromosome present in the assembly (102,883,511 bp, shown in red). Orange and pale-orange arcs show the N50 and N90 chromosome lengths (84,636,641 and 355,752 bp), respectively. The pale grey spiral shows the cumulative chromosome count on a log scale with white scale lines showing successive orders of magnitude. The blue and pale-blue area around the outside of the plot shows the distribution of GC, AT and N percentages in the same bins as the inner plot. A summary of complete, fragmented, duplicated and missing BUSCO genes in the diptera_odb10 set is shown in the top right. An interactive version of this figure is available at https://blobtoolkit.genomehubs.org/view/Anopheles funestus/dataset/CALSEJ01/snail.

The final assembly has a total length of 251 Mb in 330 sequence scaffolds with a scaffold N50 of 84.637 Mb ( Table 1). 92.38% of the assembly sequence was assigned to three chromosomal-level scaffolds, representing two autosomes (numbered and oriented against the AfunF3 assembly ( 9; GCA_003951495.1)), and the X sex chromosome ( Figure 1– Figure 4; Table 2). Synteny analysis against the AfunF3 assembly revealed multiple inversions and translocations ( Figure 5), correspondence of four largest inversions to known polymorphic inversions in *Anopheles funestus* was revealed based on population genomics ^10^ and cytogenetics ^11^ data ( Table 3).

**Table 1.** Genome data for *Anopheles funestus*, idAnoFuneDA-416_04.

*Project accession data*	
Assembly identifier	idAnoFuneDA-416_04
Species	*Anopheles funestus*
Specimen	idAnoFuneDA-416_04
NCBI taxonomy ID	62324
BioProject	PRJEB53265
BioSample ID	ERS10527360
Isolate information	female, whole organism
*Raw data accessions*	
PacificBiosciences SEQUEL I	ERR9439501
10X Genomics Illumina	ERR9356795, ERR9356796, ERR9356797, ERR9356798
Hi-C Illumina	ERR9356794
*Genome assembly*	
Assembly accession	GCA_943734845
*Accession of alternate * *haplotype*	GCA_943734645
Span (Mb)	250,713
Number of contigs	349
Contig N50 length (Mb)	24.105
Number of scaffolds	330
Scaffold N50 length (Mb)	84.637
Longest scaffold (Mb)	102.883
BUSCO * genome score	97.6

* BUSCO scores based on the diptera_odb10 (3285) set using $BUSCO_VERSION. C= complete [S= single copy, D=duplicated], F=fragmented, M=missing, n=number of orthologues in comparison. A full set of BUSCO scores is available at https://blobtoolkit.genomehubs.org/view/Anopheles%20funestus/dataset/CALSEJ01/busco.

**Figure 2.**
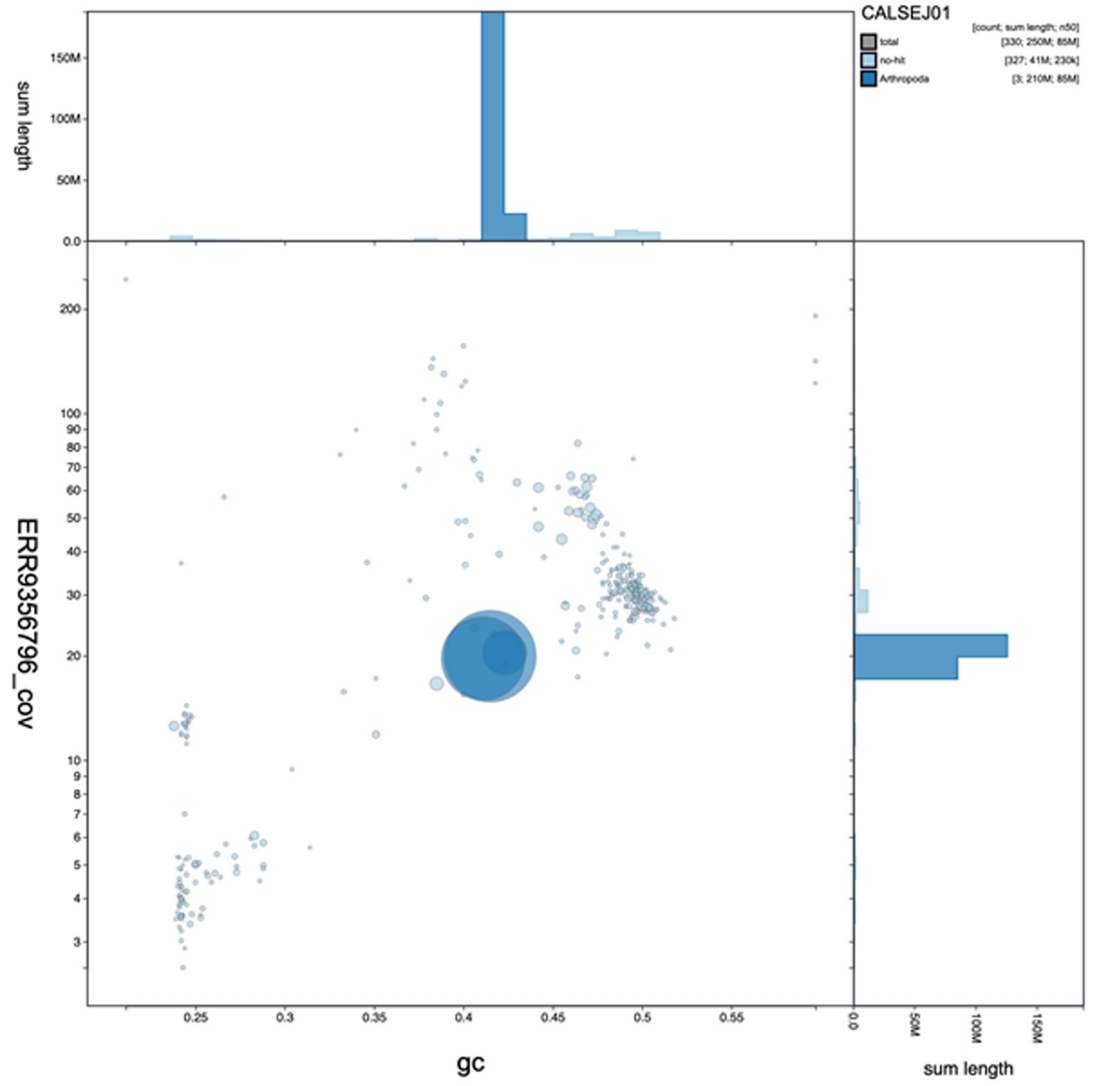
Genome assembly of *Anopheles funestus*, idAnoFuneDA-416_04: GC coverage. BlobToolKit GC-coverage plot. An interactive version of this figure is available at https://blobtoolkit.genomehubs.org/view/Anopheles funestus/dataset/CALSEJ01/blob#Filters.

**Figure 3.**
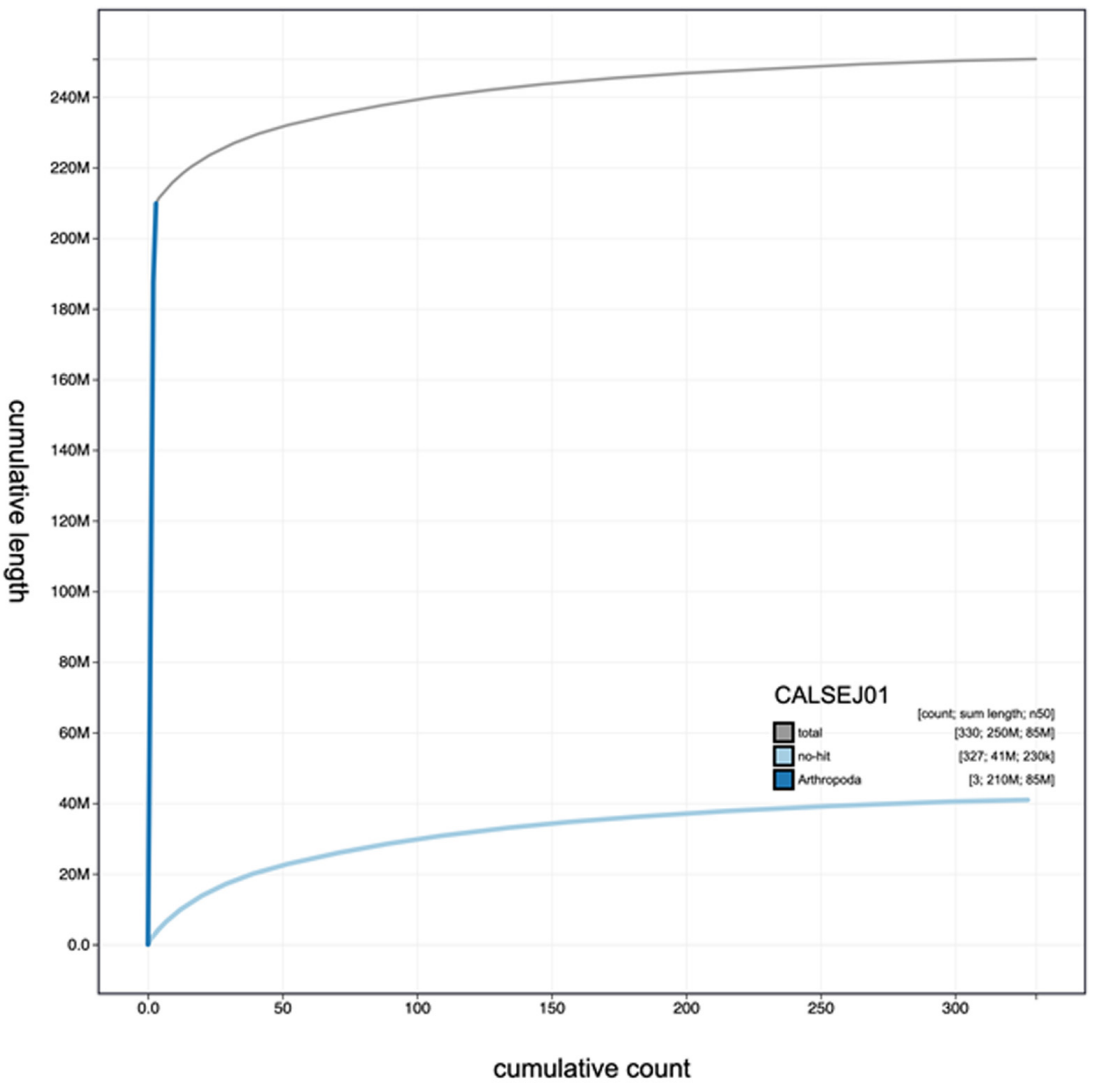
Genome assembly of *Anopheles funestus*, idAnoFuneDA-416_04: cumulative sequence. BlobToolKit cumulative sequence plot. An interactive version of this figure is available at https://blobtoolkit.genomehubs.org/view/Anopheles funestus/dataset/CALSEJ01/cumulative#Filters.

**Figure 4.**
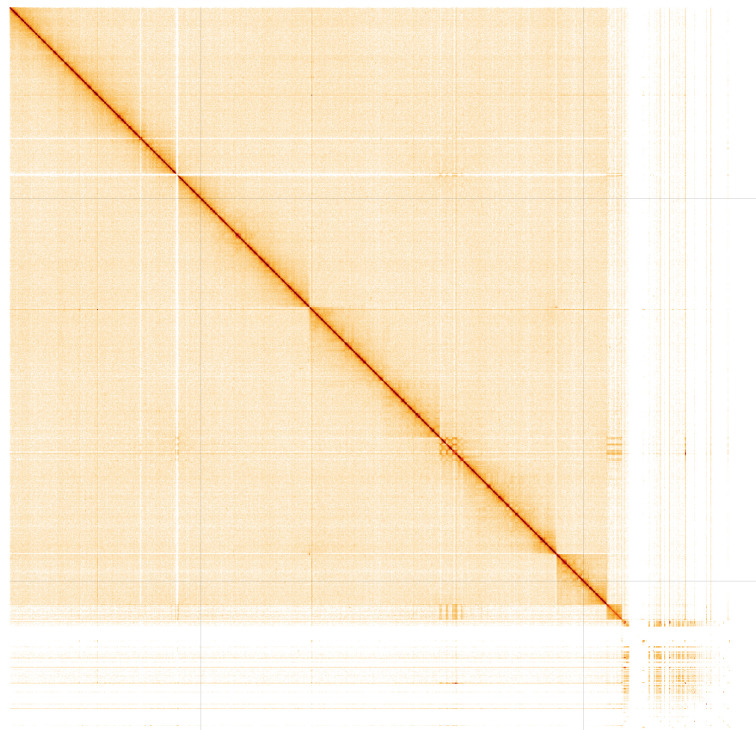
Genome assembly of *Anopheles funestus*, idAnoFuneDA-416_04: Hi-C contact map. Hi-C contact map of the idAnoFuneDA- 416_04 assembly, visualised in HiGlass. Chromosomes are arranged in size order from left to right and top to bottom. The interactive Hi-C map can be viewed at https://genome-note-higlass.tol.sanger.ac.uk/l/?d=aJmC2VieTlCIjrBC-4LXsA.

**Table 2.** Chromosomal pseudomolecules in the genome assembly of *Anopheles funestus*, idAnoFuneDA-416_04.

INSDC accession	Chromosome	Size (Mb)	Gaps
**OX030923.1**	**2RL**	**102.884**	**3**
**OX030924.1**	**3RL**	**84.637**	**4**
**OX030925.1**	**X**	**22.264**	**2**

**Figure 5.**
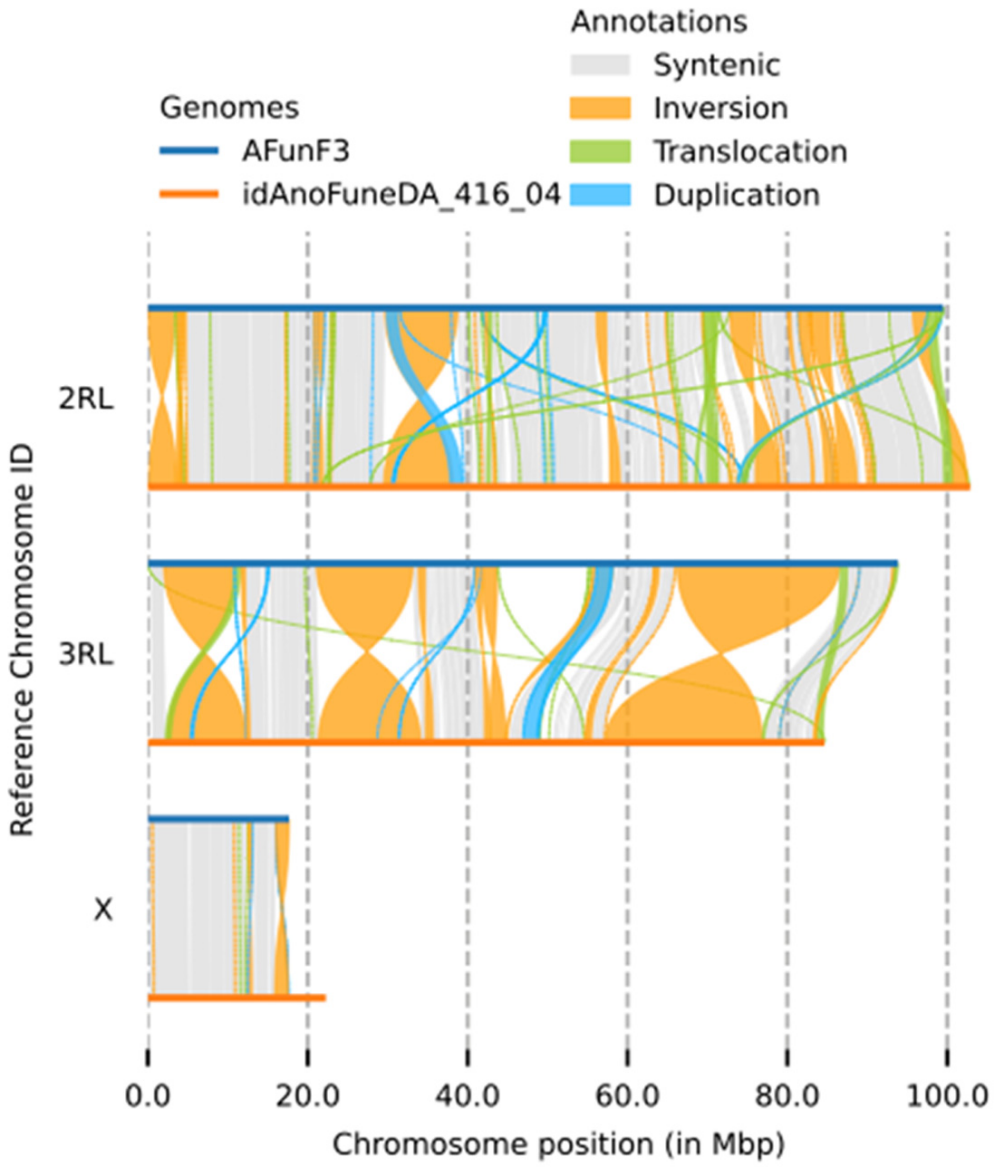
Synteny between genome assemblies of *Anopheles funestus*, AfunF3 and idAnoFuneDA-416_04. Four largest inversions identified as 2Rh, 3Ra, 3Rb, and 3La ( Table 3).

**Table 3.** Known large-scale inversions between genome assemblies of *Anopheles funestus*, AfunF3 and idAnoFuneDA-416_04 identified by syri. Coordinates given for AfunF3 (ref) and idAnoFuneDA-416_04 (q).

Inversion	Chromosome	Start in ref	End in ref	Start in q	End in q
**2Rh**	**2RL**	**29671756**	**38731257**	**29517507**	**38117412**
**3Ra**	**3RL**	**1995204**	**11122881**	**2428547**	**12234590**
**3Rb**	**3RL**	**21099051**	**33133207**	**21361107**	**34095918**
**3La**	**3RL**	**66185724**	**86544782**	**57224763**	**76848507**

The assembly has a BUSCO 5.3.2 ^12^ completeness of 97.6% using the diptera_odb10 reference set. While not fully phased, the assembly deposited is of one haplotype. Contigs corresponding to the second haplotype have also been deposited.

## Methods

### Sample acquisition and nucleic acid extraction

*Anopheles funestus* offspring were reared from a wild caught gravid female collected from La Lopé, Gabon (latitude -0.187, longitude 11.611) by Ousman Akone-ella. A single female idAnoFuneDA-416_04 was used for Pacific BioSciences and 10x genomics, its sibling female idAnoFuneDA-416_06 was used for Arima Hi-C.

For the high molecular weight (HMW) DNA extraction for *Anopheles* mosquitoes, one whole insect was disrupted by manual grinding with a blue plastic pestle in Qiagen MagAttract lysis buffer and then extracted using the Qiagen MagAttract HMW DNA extraction kit with two minor modifications. These modifications include using half volumes of the kit recommendations due to small sample size ( *Anopheles* mosquitoes typically weigh 2–3 mg) and running two elutions of 100 μl each to increase DNA yield. The quality of the DNA was evaluated using an Agilent FemtoPulse to ensure that most DNA molecules were larger than 30 kb, and preferably > 100 kb. Single mosquito extractions ranged in total estimated DNA yield from 192 ng to 800 ng, with an average yield of 500 ng. Low molecular weight DNA was removed from using an 0.8X AMpure XP purification. A small aliquot (<~5% of the total volume) of HMW DNA was set aside for 10X Linked Read sequencing and the rest of the DNA was sheared to an average fragment size of 12–20 Kb using a Diagenode Megaruptor 3 at speeds ranging from 27 to 30. Following shearing, samples were cleaned using a SPRI clean up sometimes with a bead ratio intended to help remove fragments below 3 kb. These sheared, cleaned DNAs were then evaluated again on the FemtoPulse and with PicoGreen. The median DNA fragment size was 15 kb and the median concentration of sheared DNA was 200 ng, with samples typically losing about 50% of the original estimated DNA quantity through the process of shearing and purification.

Sheared DNA was purified using AMPure PB beads with a 1.8X ratio of beads to sample to remove the shorter fragments and concentrate the DNA sample. The concentration of the sheared and purified DNA was assessed using a Nanodrop spectrophotometer and Qubit Fluorometer and Qubit dsDNA High Sensitivity Assay kit. Fragment size distribution was evaluated by running the sample on the FemtoPulse system once more on the sheared and cleaned sample.

For Hi-C samples, a separate sibling whole insect specimen idAnoFuneDA-416_06 was used as input material for the Arima V2 Kit according to the manufacturer’s instructions for animal tissue. This approach of using a sibling was taken in order to enable all material from a single specimen to contribute to the PacBio data generation given we were not always able to meet the minimum suggested guidance of starting with > 300 ng of HMW DNA from a specimen. Samples proceeded to the Illumina library prep stage even if they were suboptimal (too little tissue) going into the Arima reaction.

To assist with annotation, which will be made available through VEuPathDB Vectorbase in due course, RNA was extracted from separate whole unrelated insect specimens idAnoFuneDA-146_02, idAnoFuneDA-367_03, and idAnoFuneDA-367_04 using TRIzol, according to the manufacturer’s instructions. RNA was then eluted in 50 μl RNAse-free water and its concentration assessed using a Nanodrop spectrophotometer and Qubit Fluorometer using the Qubit RNA Broad-Range (BR) Assay kit. Analysis of the integrity of the RNA was done using Agilent RNA 6000 Pico Kit and Eukaryotic Total RNA assay. Samples were not always ideally preserved for RNA, so qualities varied but all were sequenced anyway.

### Sequencing

We prepared libraries as per the PacBio procedure and checklist for SMRTbell Libraries using Express TPK 2.0 with low DNA input. Every library was barcoded to support multiplexing. Final library concentrations ranged from 20 ng to 100 ng, and yields were typically only about 25% of the input sheared DNA. Libraries from two specimens were typically multiplexed on a single 8M SMRT Cell. Sequencing complexes were made using Sequencing Primer v4 and DNA Polymerase v2.0. Sequencing was carried out on the Sequel II system with 24 hour run time and 2 hour pre-extension. A 10X Genomics Chromium read cloud sequencing library was also constructed according to the manufacturer’s instructions (this product is no longer available). Only 0.5ng of DNA was used and only 25–50% of the gel emulsion was put forward for library prep due to the small genome size. For Hi-C data generation, following the Arima HiC 2 reaction, samples were processed through Library Preparation using a NEB Next Ultra II DNA Library Prep Kit and sequenced aiming for 100x depth. RNA libraries were created using the directional NEB Ultra II stranded kit. Sequencing was performed by the Scientific Operations core at the Wellcome Sanger Institute on Pacific Biosciences SEQUEL II (HiFi), Illumina NovaSeq 6000 (10X and Hi-C), or Illumina HiSeq 4000 (RNAseq).

### Genome assembly

Assembly was carried out with Hifiasm ^13^; haplotypic duplication was identified and removed with purge_dups ^14^. One round of polishing was performed by aligning 10X Genomics read data to the assembly with longranger align, calling variants with freebayes ^15^. The assembly was then scaffolded with Hi-C data ^16^ using SALSA2 ^17^. The assembly was checked for contamination as described previously ^18^. Manual curation was performed using gEVAL ^19^, HiGlass ^20^ and Pretext. The mitochondrial genome was assembled using MitoHiFi ^21^, which performs annotation using MitoFinder ^22^. The genome was analysed and BUSCO scores generated within the BlobToolKit environment ^23^. Synteny analysis was performed with syri v1.6 ^24^ and visualised with plotsr 0.5.3 ^25^. Table 4 contains a list of all software tool versions used, where appropriate.

**Table 4.** Software tools used.

Software tool	Version	Source
hifiasm	0.14	13
purge_dups	1.2.3	14
SALSA2	2.2- 4c80ac1	17
longranger align	2.2.2	https://support.10xgenomics.com/ genome-exome/software/pipelines/ latest/advanced/other-pipelines
freebayes	1.3.1	15
MitoHiFi	2	21
gEVAL	N/A	19
HiGlass	1.11.6	20
PretextView	0.1.x	https://github.com/wtsi-hpag/ PretextView
BlobToolKit	2.6.2	23

### Ethics/compliance issues

The genetic resources accessed and utilised under this project were done so in accordance with the UK ABS legislation (Nagoya Protocol (Compliance) (Amendment) (EU Exit) Regulations 2018 (SI 2018/1393)) and the national ABS legislation within the country of origin, where applicable.

## Data Availability

European Nucleotide Archive: *Anopheles funestus* genome assembly, idAnoFuneDA-416_04. Accession number PRJEB53266; https://identifiers.org/bioproject/PRJEB53266. The genome sequence is released openly for reuse. The *Anopheles funestus* genome sequencing initiative is part of the Anopheles Reference Genomes project. All raw sequence data and the assembly have been deposited in INSDC databases. Raw data and assembly accession identifiers are reported in Table 1.

## Author information

Members of the Sanger Scientific Operations: DNA Pipelines collective are listed here: https://doi.org/10.5281/zenodo.4790456.
