# Supplementary material for: The genome sequence of the malaria mosquito, Anopheles funestus, Giles, 1900
Source: Wellcome Open Res. 2023 Mar 27;7:287. Originally published 2022 Nov 25. [Version 2] doi: 10.12688/wellcomeopenres.18445.2 (PMC9975407; doi:10.12688/wellcomeopenres.18445.2)
Supplement: Peer review history

# Peer review: Reviewer response for version 1

**DOI:** 10.21956/wellcomeopenres.20454.r53476

**Published:** 2022-12-21

**Authors:** Ben Matthews

**Affiliations:** 1 Department of Zoology, University of British Columbia, Vancouver, BC, Canada

In their data note entitled: “The genome sequence of the malaria mosquito, *Anopheles funestus*, Giles, 1900,” Ayala and colleagues clearly describe the generation of a PacBio long read + 10X genomics genome assembly from a single female offspring from a single wild female mosquito collected in La Lopé, Gabon, with additional scaffolding provided from a sibling via Hi-C. The resulting assembly provides a marked improvement over the previous reference genome, though reveals some discrepancies in the form of inversions and other structural rearrangements that will be important to dig into.

Rationale:

*Anopheles funestus* is a major malaria vector and thus any information about its biology is critically important for control efforts. A complete and contiguous reference genome is an important piece for a variety of research surrounding the biology of this deadly vector.

Protocols appropriate, and work technically sound:

 The collection, HMW DNA extraction, DNA QC, library prep, sequencing, and assembly informatics are clearly described and appropriate. The resulting assembly is technically sound given current state-of-the-art assembly.

Sufficient details of methods and materials:

 Generally very clear, with one exception. On page four of the methods, the following sentence is vague: “Following shearing, samples were cleaned using a SPRI clean up sometimes with a bead ratio intended to help remove fragments below 3 kb.” In particular, the word sometimes here is confusing - for the assembly in question, was a SPRI clean up used to remove fragments below 3kb? If this approach is used sometimes, how does one decide when and when not to apply it? What is the bead ratio? This information is required to make the methods complete.

Datasets clearly presented in useable and accessible format:

 Table 1 and links provided within the paper provide complete access to the datasets and raw sequencing reads in standard formats at standard repositories.

Are sufficient details of methods and materials provided to allow replication by others?

Yes

Is the rationale for creating the dataset(s) clearly described?

Yes

Are the datasets clearly presented in a useable and accessible format?

Yes

Are the protocols appropriate and is the work technically sound?

Yes

Reviewer Expertise:

Mosquito genomics, behaviour, neuroscience

I confirm that I have read this submission and believe that I have an appropriate level of expertise to confirm that it is of an acceptable scientific standard.

# Peer review: Reviewer response for version 1

**DOI:** 10.21956/wellcomeopenres.20454.r53455

**Published:** 2022-12-19

**Authors:** Igor V Sharakhov

**Affiliations:** 1 Department of Entomology, Virginia Polytechnic Institute and State University, Blacksburg, USA

The article by Ayala and coauthors present a new genome assembly obtained from an individual female *Anopheles funestus*. The genome was sequenced using the PacBio procedure, polished by 10X Genomics Chromium reads, and scaffolded into three chromosomal pseudomolecules using Hi-C data obtained from a single sibling mosquito. The complete mitochondrial genome was also assembled. The rationale for creating the *Anopheles funestus* genome is clearly described. The previous genome assembly originated from a colony derived from wild individuals, while the new genome assembly is obtained from a single mosquito and is of higher quality. The protocols are appropriate and the work is technically sound. The manuscript provides sufficient details of methods and materials to allow replication by others. The datasets are clearly presented in a useable and accessible format.

 Minor comments:

- The Fumoz genome assembly was improved not just by using long read sequencing but also by Hi-C.
- In Table 1, Assembly accession and Accession of alternate haplotype numbers are mixed up here or on the NCBI website.
- “The median DNA fragment size was 15 kb and the median concentration of sheared DNA was 200 ng,” change to “The median DNA fragment size was 15 kb and the median amount of sheared DNA was 200 ng,”
- Label chromosome arms in Figure 4.
- A reference is missing for the Pretext software.
- Figure 5 shows a number of small inversions, duplications and translocations, which could be misassemblies in one of two genomes. Please indicate methods of validation of genomic arrangements. 

Are sufficient details of methods and materials provided to allow replication by others?

Yes

Is the rationale for creating the dataset(s) clearly described?

Yes

Are the datasets clearly presented in a useable and accessible format?

Yes

Are the protocols appropriate and is the work technically sound?

Yes

Reviewer Expertise:

Mosquito evolutionary genetics, cytogenetics, genomics

I confirm that I have read this submission and believe that I have an appropriate level of expertise to confirm that it is of an acceptable scientific standard.
